# Protein-binding Properties of two Antitumour Ru(III) Complexes to Human Apotransferrin and Apolactoferrin

**DOI:** 10.1155/MBD.1994.169

**Published:** 1994

**Authors:** F. Kratz, B. K. Keppler, L. Messori, C. Smith, E. N. Baker

**Affiliations:** 1 Department of Inorganic Chemistry University of Heidelberg Im Neuenheimer Feld 270 Heidelberg W - 6900 Germany; 2 Massey University Department of Chemistry and Biochemistry Palmerston North New Zealand

## Abstract

The interaction of two ruthenium(III) complexes exhibiting high anticancer activity - namely *trans*-Indazolium(bisindazole) tetrachlororuthenate(III), Hlnd[RuInd_2_Cl_4_], and *trans*-Imidazolium (bisimidazole) tetrachlororuthenate(III), Hlm[RuIm_2_Cl_4_], - with human serum apotransferrin has been investigated through spectroscopic and chromatographic techniques with the ultimate goal of preparing adducts with good selectivity for cancer cells due to the fact that tumour cells express high amounts of transferrin receptors on their cell surface. Whereas the binding of Hlm[RuIm_2_Cl_4_] to human serum apotransferrin takes several hours, Hlnd[RuInd_2_Cl_4_], the less toxic complex, gives rise to a well defined 2:1 complex within a few minutes. Hlnd[RuInd_2_Cl_4_] will react with apotransferrin only in the presence of bicarbonate, this anion dictating the kinetic and mechanistic characteristics of protein-binding. Circular dichroism studies had previously indicated that binding of both Ru(III) complexes occurs around the unoccupied iron(III) binding sites; this result is now confirmed by preliminary X-ray data of Hlnd[RuInd_2_Cl_4_] and Hlm[RuIm_2_Cl_4_] bound to apolactoferrin, a related iron protein. The crystallograhic data reveals that binding of both complexes takes place at histidine residues, and that the ligand (indazole) remains bound in the case of Hlnd[RuInd_2_Cl_4_].


### PROTEIN-BINDING PROPERTIES OF TWO ANTITUMOUR Ru(III) COMPLEXES TO HUMAN APOTRANSFERRIN AND APOLACTOFERRIN

F. Kratz*[1], B.K. Keppler[1], L. Messori[1], C. Smith[2] and E.N. Baker[2]

[1]Department of Inorganic Chemistry, University of Heidelberg, Im Neuenheimer Feld 270, W - 6900 Heidelberg 1, Germany
[2] Massey University,
Department of Chemistry and Biochemistry, Palmerston North, New Zealand

### Abstract

The interaction of two ruthenium(III) complexes exhibiting high anticancer activity - namely *trans*-Indazolium(bisindazole) tetrachlororuthenate(III), HInd[$RuInd_2Cl_4$], and *trans*-Imidazolium (bis-imidazole) tetrachlororuthenate(III), HIm[$RuIm_2Cl_4$], - with human serum apotransferrin has been investigated through spectroscopic and chromatographic techniques with the ultimate goal of preparing adducts with good selectivity for cancer cells due to the fact that tumour cells express high amounts of transferrin receptors on their cell surface.
Whereas the binding of HIm[$RuIm_2Cl_4$] to human serum apotransferrin takes several hours, HInd[$RuInd_2Cl_4$] , the less toxic complex, gives rise to a well defined 2:1 complex within a few minutes. HInd[$RuInd_2Cl_4$] will react with apotransferrin only in the presence of bicarbonate, this anion dictating the kinetic and mechanistic characteristics of protein-binding.
Circular dichroism studies had previously indicated that binding of both Ru(III) complexes occurs around the unoccupied iron(III) binding sites; this result is now confirmed by preliminary X-ray data of HInd[$RuInd_2Cl_4$] and HIm[$RuIm_2Cl_4$] bound to apolactoferrin, a related iron protein. The crystallograhic data reveals that binding of both complexes takes place at histidine residues, and that the ligand (indazole) remains bound in the case of HInd[$RuInd_2Cl_4$].

### Introduction

The Ru(III) complexes, HIm[$RuIm_2Cl_4$] - abbrev. ru-im - (Fig. 1A) and HInd[$RuInd_2Cl_4$] - abbrev. ru-ind - (Fig. 1B), show a high antitumour activity in the autocthonous colorectal carcinoma model of rats, a model which simulates the colon cancer of humans very well [1].

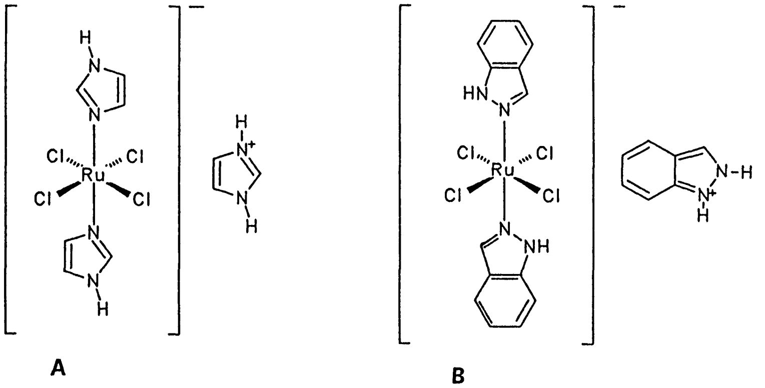

Figure 1. Scheme of the two ruthenium(III) complexes: A) ru-im; B) ru-ind.

Recently, it has been shown by Kratz et al. that the most promising complex, ru-ind, which is far less toxic in long term application than the imidazole analogue, binds within a few minutes to the serum proteins albumin and transferrin [2]. Furthermore, it has been demonstrated that the apotransferrin-bound complex exhibits a higher antitumour activity against human colon cancer cells when compared to the albumin-bound or "free" species [3]. This result suggests that apotransferrin can act as a natural carrier of the drug to the tumor tissues owing to the high affinity between this metal transport protein and the large number of transferrin receptors on the surface of tumor cells [4]. Indeed, it has been previously reported that transferrin is responsible for the selective delivery of radioactive $^{67}Ga(III)$ complexes to tumor tissues [5], and it is therefore tempting to exploit the transferrin cycle as a "natural" route for selective delivery of cytostatic drugs to cancer cells. We therefore decided to investigate the kinetic and mechanistic characteristics of the reaction between the two ruthenium(III) complexes and apotransferrin with the aid of HPLC. Furthermore, in order to obtain more precise information on the binding sites of the complexes, soaking experiments of apolactoferrin crystals with the two Ru(III) complexes were carried out and preliminary results of the subsequent X-ray structure analyses are reported in this paper.

### Materials and Methods

The ruthenium(III) complexes were synthesized as described earlier [1] and were used in all the experiments from a freshly prepared $5 \times 10^{-4}$ M aqueous solution for ru-ind and a $1 \times 10^{-2}$ M aqueous solution for ru-im. Human serum apotransferrin (98%, crystalline, essentially iron free, MW 80,000) (apoTf hereafter) were purchased from Sigma Chemical Company and the latter purified according to standard procedures [6]. For the kinetic and mechanistic studies a physiological buffer was used so that the final concentrations were 0.004 M $NaH_2PO_4$, 0.1 M NaCl and 0.025 M $NaHCO_3$ with pH=7.4.

HPLC studies were performed with a Perkin Elmer Series 410 LC pump and a LC-95 UV/visible spectrophotometer detector. The column used was: (a) Bio-Sil SEC 250, (300 mm x 7.8 mm) from Bio-RAD, mobile phase: 0.15 M NaCl, 0.01 M $NaH_2PO_4$, 5% $CH_3CN$ - pH 7.0.

Human apolactoferrin was prepared and crystallized (in deglycosylated form) as described by Norris et al. [7]. The crystals of apolactoferrin were reacted with the Ru(III) complexes by soaking them in mother liquor (0.05 M tris-buffer, pH 7.8, containing 7% ethanol and 5% 2-methyl-2,4-pentanediol) to which $NaHCO_3$ (0.01M) and ru-im or ru-ind had been added to concentrations of 0.01 M and 0.0005 M respectively. Soaking times between 12 hours and 4 weeks were used after which the crystals were removed and mounted in glass capillaries. X-ray data were collected with a Rigaku R-axis image plate detector on a rotating anode generator to a resolution of 2.2 Å.

### Results and Discussion

### *Kinetics and mechanism of protein-binding*

The reactivity pattern of both ru-ind and ru-im with apotransferrin is complicated by the occurence of concomitant hydrolysis processes and substitution reactions with solute species. Surprisingly, in spite of their structural similarity, ru-im and ru-ind exhibit very different reactivity patterns which are reflected in the respective protein-binding abilities.

The major differences between the behavior of the two complexes is a kinetic and mechanistic one. Binding of ru-ind to apotransferrin proceeds through the formation of two intermediates which then bind rapidly to the protein as can be seen in the respective chromatograms (Fig.2 A-C).

Fig. 2A shows a chromatogram of ru-ind by itself, it can be clearly seen that the complex anion $[RuInd_2Cl_4]^-$ (12.5 min) is separated from the cation $[HInd]^+$ (15.8 min). Fig. 2B shows the chromatogram of ru-ind and apotransferrin after only 3 min of incubation. The original complex (12.5 min) has disappeared, but two intermediate complexes appear (peaks at 10.3 and 11 minutes), and there is already binding to apotransferrin (5.5 min). After 5 minutes (Fig 2C) the reaction is complete, all of the complex now being bound to apotransferrin. The important thing to note is that this reaction takes place only in the presence of bicarbonate. This is a novel feature of this ruthenium(III) complex and implies that it is not simple hydrolysis but that a number of substitution reactions involving bicarbonate are taking place before binding to apotransferrin.

In contrast, it takes 5 hours at 37 $^{o}C$ for ru-im to bind to apotransferrin; the reaction does not depend on bicarbonate, and no intermediates are observed in the chromatograms (Fig. 3A,B).

Fig. 3A shows the reaction of ru-im and apotransferrin after 3 minutes followed at 254 nm and 345

Figure 2A-C : Chromatograms of: A) ru-ind, B) ru-ind plus apoTf after 3 minutes, C) ru-ind plus apoTf after 5 minutes. All chromatograms were performed on a size exclusion column and recorded at 280 and 360 nm. ru-ind was incubated with apoTf in the physiological buffer at T = 37 $^{o}$C at a ratio of 1:1, the concentration of apoTf being $1x10^{-4}$ M. The peak of apoTf is labelled T.

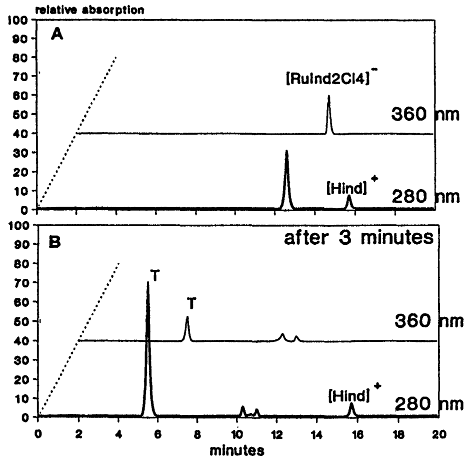

Figure 3A,B: Chromatograms of: A) ru-im plus apoTf after 3 minutes, B) ru-im plus apoTf after 2 hours. The chromatograms were performed on a size exclusion column and recorded at 254 and 340 nm. ru-im was incubated with apoTf in the physiological buffer at T = 37 $^{o}$C at a ratio of 1:1, the concentration of apoTf being $5x10^{-4}$M. The peak of apoTf is labelled A.

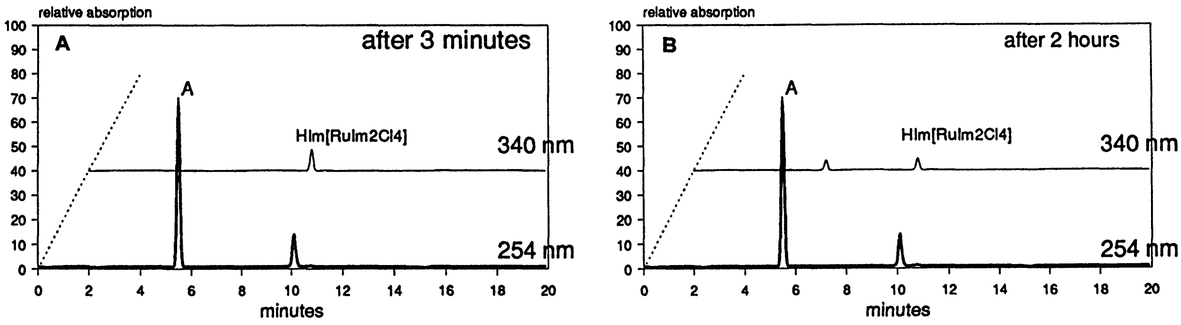

nm (LMCT): only one peak is seen for ru-im at 10 min suggesting that the complex exists in solution as a stable ionic pair. Another thing which can be observed is that after this short time interval there is neither binding to apotransferrin nor detection of intermediates. Fig. 3B shows the chromatogramm of ru-im and apotransferrin after 2 hours incubation. The original complex is still present although the signal intensity is reduced to about one half of the original value, and a clear signal at 5.5 min, the position of apotransferrin, is now observed at 340 nm. A further thing to note is that no signal for a free imidazolium ion appears in the chromatogramm indicating that the original imidazolium (bisimidazole)tetrachlororuthenate complex might bind to apotransferrin as a tris-imidazole complex. Protein-binding is complete after approximately 5 hours when the chromatogram shows only the apotransferrin peak at both wavelengths.

*Specificity of protein-binding and crystallographic studies*

We have previously shown by circular dichroism (CD) spectroscopy that 2 equivalents of ru-ind can be specifically bound by apotransferrin indicating that binding takes place around the two iron(III) binding sites of the protein (2).

In order to gain more definitive information on the binding sites for the two Ru(III) complexes, crystallographic experiments were carried out. Apolactoferrin was chosen rather than apotransferrin because of the availability of suitable crystals, but the binding behaviour should be similar given the strong structural similarities between lactoferrins and transferrins (8). The analyses carried out to date include one in which a crystal of apolactoferrin was soaked in ru-ind for a period of 12 hours, and a second in which ru-im was used for a much longer period of 4 weeks. Although the results are preliminary only, as full crystallographic refinements have not yet been carried out, difference Fourier maps show very clearly that the preferred binding sites for the Ru(III) complexes are at histidine residues of the protein, presumably following the loss of one or more of the chloride ligands. In the case of ru-ind, with a short soaking time and a low concentration, binding occurs specifically at His 253 in the open binding cleft of the N-terminal half of the protein; this residue is one of the iron ligands in the diferric form of lactoferrin or transferrin (8). The two indazole ligands remain coordinated to the ruthenium atom (Fig. 4a).

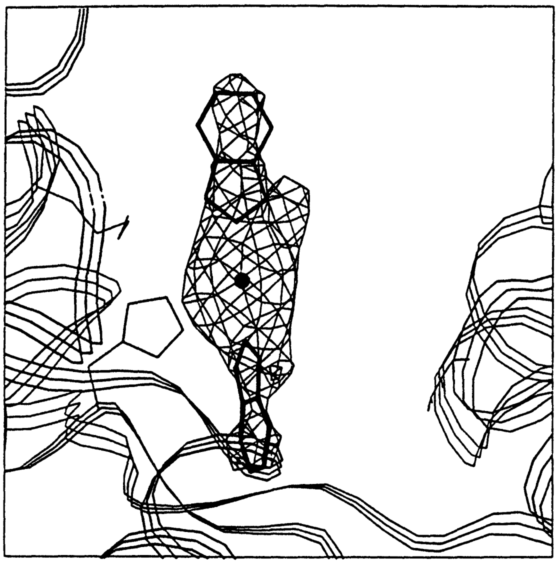

Figure 4a: Difference electron density for ru-ind in the N-terminal site of human apo-lactoferrin, showing that the two indazole ligands are retained. The Ru atom binds to His 253, and the nearby side chain of Lys 301 may help stabilize binding.

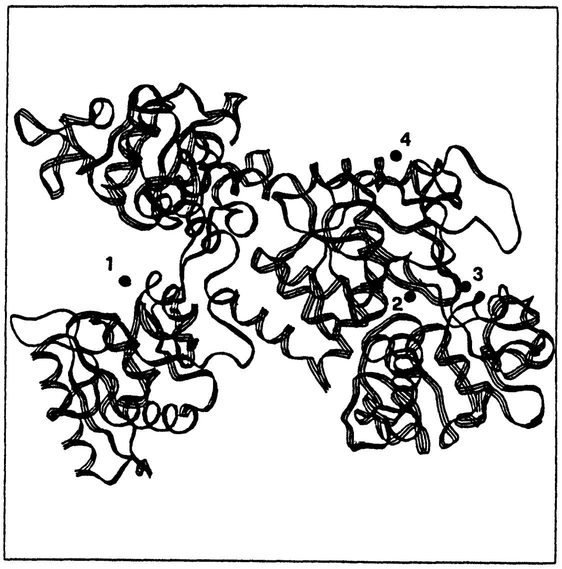

Figure 4b: Ribbon diagram showing sites of ru-im binding after soaking for 4 weeks. Sites are: (1) His 253, (2) His 597, (3) His 590, (4) His 654.

When ru-im is used, with the higher concentration and longer soaking time, more sites are occupied (Fig. 4b). These include the histidine ligands at both iron binding sites, His 253 and His 597, as well as other non-specific sites where histidines are exposed on the protein surface, i.e. His 590 and His 654. In these cases the preliminary nature of the results is such that we cannot yet say to what extent the imidazole ligands remain bound.

*Acknowledgments.*
This work has been supported by the Dr. Mildred Scheel Stiftung der Deutschen Krebshilfe, FRG.

**References**

1. Keppler, B.K., Henn, M., Juhl, U.M., Berger M.R., Niebl R., Wagner F.F. (1989) *Prog. Clin. Biochem. Med., vol.10,* 41-69, Springer Verlag, Berlin Heidelberg.
2. Kratz, F., Mulinacci, N., Messori, L., Bertini, I., Keppler, B.K. (1992) *Metal Ions in Biology and Medicine, vol. 2*, 69-74, John Libbey Limited Eurotext, Paris.
3. Berger, M.R., Keppler, B.K., Messori, L., and Kratz, F. (1993) manuscript in preparation.
4 Brock, J.H. (1985): Transferrins, 183-263, *in Metalloproteins, Part 2*, ed. P.M. Harrison, VCH, Weinheim.
5. Ward, S.G. and Taylor, R.C. (1988) *Metal based Antitumor Drugs* (Gielen, M.F., ed.) Freund Publishing House Ltd., chapter 1.
6. Schlabach, M.R., Bates, G.W. (1975) *J. Biol. Chem. 250*, 2177-2182
7. Norris, G.E., Baker, H.M. and Baker, E.N., (1989), *J. Biol. Chem. 209*, 329-331.
8. Baker, E.N. and Lindley, P.F. (1992), *J. Inorg. Biochem. 47 (3-4)*, 147-160.

**Received: September 2, 1993**

